# Sedentary and 21 gastrointestinal disorders: A Mendelian randomization study

**DOI:** 10.1097/MD.0000000000039813

**Published:** 2024-09-20

**Authors:** Yunzhi Lin, Jun He, Zhen Ding

**Affiliations:** a Department of Hepatobiliary Surgery, Chaohu Hospital of Anhui Medical University, Hefei, China.

**Keywords:** Gastrointestinal diseases, Mendelian randomization, sedentary, single nucleotide polymorphisms, watching television

## Abstract

Sedentary behavior (SB) has been linked in the past by observational studies to gastrointestinal illnesses, although the exact cause of the link is still unknown. To deal with this problem, we carried out a Mendelian randomization (MR) study to thoroughly examine the connection between SB and common gastrointestinal illnesses. We selected instrumental variables representing the SB from the UK Biobank study, including watching television viewing, playing computer, and driving. In addition, we obtained genetic associations of 21 common gastrointestinal disorders from the FinnGen research. After adjusting for common risk factors associated with gastrointestinal diseases, we analyzed the independent association between genetic. Furthermore, we used the inverse-variance weighted (IVW) method in conjunction with complementing techniques like MR-Egger (Mendelian randomization based on Egger Regression) and weighted median to assure the accuracy and dependability of the results. Our findings suggest that genetic susceptibility to prolonged television viewing is significantly associated with an increased risk of 9 out of 21 gastrointestinal disorders. Specifically, these disorders include gastroesophageal reflux disease, chronic gastritis, cholelithiasis, acute pancreatitis, chronic pancreatitis, gastroduodenal ulcer, fatty liver, irritable bowel syndrome, and acute appendicitis. These associations remained significant even after correcting for potential confounding factors. The replication analysis confirms the same conclusion. The results of this study demonstrate a causal relationship between cachexia and genetically predicted SB. To further understand the underlying pathogenic mechanisms at play, more study is required.

## 1. Introduction

Any activity with an intensity of <1.5 metabolic equivalents is considered sedentary behavior (SB), which has a variety of negative effects on human health.^[1,2]^ The sedentary lifestyle, which includes a variety of activities like watching television, using computers, and driving, is quickly becoming prevalent throughout the world.^[3,4]^ A growing number of research have shown that SB has a significant impact on human health, indicating that there has been tremendous progress in the study of SB and its effects on health. As a result, providing appropriate advice to limit inactive time may help in lowering the incidence of diseases, a global public health concern that is on the rise.^[1,2,5–8]^

The relationship between sedentary time and cognitive function in older individuals was explored in a cross-sectional research of older persons from Japan. The study proved that extended periods of inactivity were detrimental to one’s capacity to orient oneself and that this association was held even after controlling for potential confounding variables like moderate to strenuous physical exercise. This result implies that decreasing sedentary time may benefit older persons’ cognitive function.^[9]^ Reducing the amount of time people spend watching television may be a goal for public health interventions, as evidenced by a large prospective American study demonstrating long-term television viewing is significantly linked to the risk of 8 different forms of death.^[10]^ A different investigation involving 430,584 participants from the UK Biobank discovered that SB was linked to a higher risk of colorectal cancer and that cutting out on sedentary time may help to lower this risk.^[11]^ Furthermore, a review of the National Association of Retired Persons Diet and Health Study revealed a slight association between SB and stomach and esophageal cancers.^[12]^

Although earlier studies have shown that SB is a risk factor for gastrointestinal disorders, it is critical to recognize that confounding factors and the possibility of reverse causal relationships cannot be discounted. These studies frequently have intrinsic shortcomings as well. As a result, it is unclear if there is a causative link between SB and gastrointestinal disorders and what specific function it plays in aggravating these problems. Mendelian Randomization (MR) analysis, leveraging the comprehensive Genome wide association study (GWAS) database, offers a robust approach to address these complexities. By taking into account allele distribution randomization during gamete production, MR performs similarly to randomized controlled trials and is in accordance with Mendel’s second rule of genetics. This technique uses genetic variants as instrumental factors to examine causal relationships between illness phenotypes and phenotypes. Notably, it endeavors to mitigate potential confounders and biases stemming from reverse causal relationships, thereby enhancing the credibility of findings to a significant extent.^[13,14]^

MR procedures have advanced significantly as a result of the recent creation of large-scale GWAS databases, which provide associated genetic variants and single nucleotide polymorphisms (SNPs) through DNA and whole genome sequencing. While previous MR studies have explored the link between recreational SB and certain diseases,^[15,16]^ the relationship between SB and gastrointestinal diseases has been less explored. In this study, we aim to fill this gap by investigating the causal association between SB and 21 gastrointestinal diseases using 3 types of leisurely SBs (watching television, playing on the computer, and driving) as exposures. Through MR analysis methods using existing GWAS datasets, we aim to provide further evidence to support global public health efforts.

## 2. Materials and methods

To establish a causal connection, MR analysis makes 3 assumptions instrumental variables must have a strong correlation with SB, instrumental variables can only affect gastrointestinal diseases through SB, and instrumental variables cannot be influenced by other confounding factors in the occurrence and progression of gastrointestinal diseases.^[13,17]^ Public GWAS databases will be used as the data source in this study, and all available data will be obtained and used. No new ethical approval is needed for this investigation because all participants in the original studies gave their informed consent and got ethical approval from the relevant ethics committee.

## 3. SB data sources

A total of 437,887, 360,895, and 310,555 samples were included in this study, utilizing GWAS data from the UK Biobank database as genetic predictive instrumental variables for TV watching, computer playing, and driving (Table 1). The UK Biobank is a large prospective research cohort that enrolled approximately half a million subjects from 2006 to 2010 to identify the causes of disease through self-report, medical records, and follow-up studies to benefit global human health.^[18]^ Using Wang et al’s GWAS meta-analysis of leisure screen time for 526,725 Europeans as a validation group.^[15]^ To access the corresponding instrumental variables, we applied a gene-wide significance threshold of *P* < 5E−08, while also removing the effect of linkage disequilibrium (clump *r*^2^ < 0.001 and clump distance > 100,00kb). In cases where the number of screened SNPs was less than 3, we adjusted the threshold to *P* < 5E−06. Ultimately, we identified 113, 83, and 59 SNPs from the GWAS dataset that represented SB as instrumental variables for TV watching, computer playing, and driving time, respectively. Additionally, we removed SNPs with inverted repeat sequences to ensure that the effects of these SNPs on exposure corresponded to the same alleles as the effects.

**Table 1:** Sources of exposure and outcome data.

Exposure/outcome	Cases	Controls/sample size	Data sources
Time spent watching television	–	437,887	https://gwas.mrcieu.ac.uk/
Leisure screen time	–	526,725	https://www.ebi.ac.uk/gwas/
Time spent using computer	–	360,895	https://gwas.mrcieu.ac.uk/
Time spent driving	–	310,555	https://gwas.mrcieu.ac.uk/
Body mass index	–	339,224	https://portals.broadinstitute.org/collaboration/giant
Cigarettes smoked per day	–	249,752	https://genome.psych.umn.edu/index.php/GSCAN
Type 2 diabetes	–	655,666	http://cnsgenomics.com/data.html
Gastroesophageal reflux	22,867	292,256	https://r8.finngen.fi/
Esophageal cancer	503	259,583	https://r8.finngen.fi/
Acute gastritis	2120	292,256	https://r8.finngen.fi/
Chronic gastritis	8621	292,256	https://r8.finngen.fi/
Gastroduodenal ulcer	8240	292,256	https://r8.finngen.fi/
Gastric cancer	1227	259,583	https://r8.finngen.fi/
Fatty liver	1908	340,591	https://r8.finngen.fi/
Liver fibrosis	130	338,951	https://r8.finngen.fi/
Cirrhosis	3548	338,951	https://r8.finngen.fi/
Hepatoma	648	259,583	https://r8.finngen.fi/
Cholelithiasis	34,461	301,383	https://r8.finngen.fi/
Acute pancreatitis	5509	301,383	https://r8.finngen.fi/
Chronic pancreatitis	3002	301,383	https://r8.finngen.fi/
Pancreatic cancer	1249	259,583	https://r8.finngen.fi/
Celiac Disease	3320	328,204	https://r8.finngen.fi/
Ulcerative colitis	4460	337,399	https://r8.finngen.fi/
Crohn’s disease	1484	340,875	https://r8.finngen.fi/
Irritable bowel syndrome	8116	276,683	https://r8.finngen.fi/
Acute appendicitis	25,706	314,763	https://r8.finngen.fi/
Colon cancer	3292	259,583	https://r8.finngen.fi/
Rectal cancer	2017	259,583	https://r8.finngen.fi/

## 4. Gastrointestinal disease data sources

The 21 gastrointestinal diseases used in this study were obtained from the latest version (r8) of the FinnGen database (https://r8.finngen.fi/, RRID: SCR_022254), which is a large-scale biospecimen database that employs reliable diagnostic technologies and public health systems (Table 1). The database has been collecting data from the national health registry of the Finnish population since 1969, enabling a deeper understanding of genome-health associations and facilitating the medical community’s exploration of the causes of disease in the population.^[19,20]^ We utilized GWAS data for 21 gastrointestinal diseases from the FinnGen database. The sample populations for the exposure and outcome were of European ethnic origin and showed no apparent overlap.

## 5. Analysis of MR

In the present study, we utilized MR analysis to investigate the genetically predicted causal links between leisure SBs, namely television watching, computer playing, and driving time, and 21 gastrointestinal disorders. Simultaneously using replication analysis to validate our findings. The multiplicative random effects inverse-variance weighted (IVW) method, which uses weighted linear regression and presumes that the chosen instrumental variables are independent of one another, was the primary statistical technique used. This approach is robust even in the presence of heterogeneity.^[21]^ We used IVW in conjunction with complementing techniques like Mendelian randomization based on Egger Regression (MR-Egger) and weighted median to assure the accuracy and dependability of the results.^[22–24]^ The MR-Egger approach checks the horizontal pleiotropy of instrumental variables and determines whether an intercept term is present. When the *P*-value is <.05, we consider the study results to suggest potential horizontal pleiotropy.^[25]^ Heterogeneity in the IVW method was assessed by estimating Cochran’s *Q* value, and we assume the presence of heterogeneity between instrumental variables when the *P*-value is <.05.^[26]^ We identified aberrant SNPs that affect the outcomes of the MR analysis using the MR-PRESSO tool, and then we repeated the MR analysis after eliminating those SNPs to remove any outliers among the instrumental variables.^[27]^

Additionally, we used a multivariate MR approach to evaluate the independent effects of television viewing and gastrointestinal disorders while controlling for variables frequently associated with gastrointestinal disorders (Table 1), such as body mass index (BMI), daily smoking, and type 2 diabetes, to investigate the causal association between genetically predicted television viewing and gastrointestinal disorders.^[28–31]^ In this study, we employed a 2-sample MR approach to estimate the effects of the SB on the intermediate factors. Subsequently, we used multivariable MR to adjust for SB and evaluate the impact of the intermediate factors on gastrointestinal diseases. Finally, the proportion of the mediation effect is calculated by multiplying these 2 effect estimates and dividing the result by the total effect. This comprehensive approach provides valuable insights into the mediating role of the intermediate factors between exposure and gastrointestinal diseases. Besides, we applied the Benjamin-Hochberg method to control the False Discovery Rate (FDR) for correcting multiple tests. Correlations with a corrected *P*-value < .05 using the Benjamin–Hochberg method were considered statistically significant. To avoid the bias of weak instrumental variables, we calculated the *F*-value statistic of instrumental variables by the formula β²/SE² and excluded it when the *F*-value was <10.^[32]^ Analyses were performed in R software (RRID: SCR_000432) and carried out with TwoSampleMR, MendelianRandomization, and MR-PRESSO packages.^[27,33,34]^

## 6. Results

In this study, the instrumental variables selected for analysis had F-statistics ranging from 20 to 716 for BMI, 29 to 961 for daily smoking, and 29 to 1578 for type 2 diabetes. For the time spent watching television, the *F*-statistics ranged from 30 to 151, while for the time spent playing on the computer, they ranged from 29 to 89, and for the time spent driving, they ranged from 20 to 39. LSB Instrumental variables estimation *F* statistical value is between 29 and 97. Notably, all of these instrumental variables had *F*-values >10, indicating that no bias was found for weak instrumental variables (Tables S1–S5, Supplemental Digital Content, http://links.lww.com/MD/N612).

## 7. Univariate results

**Figure 1.**
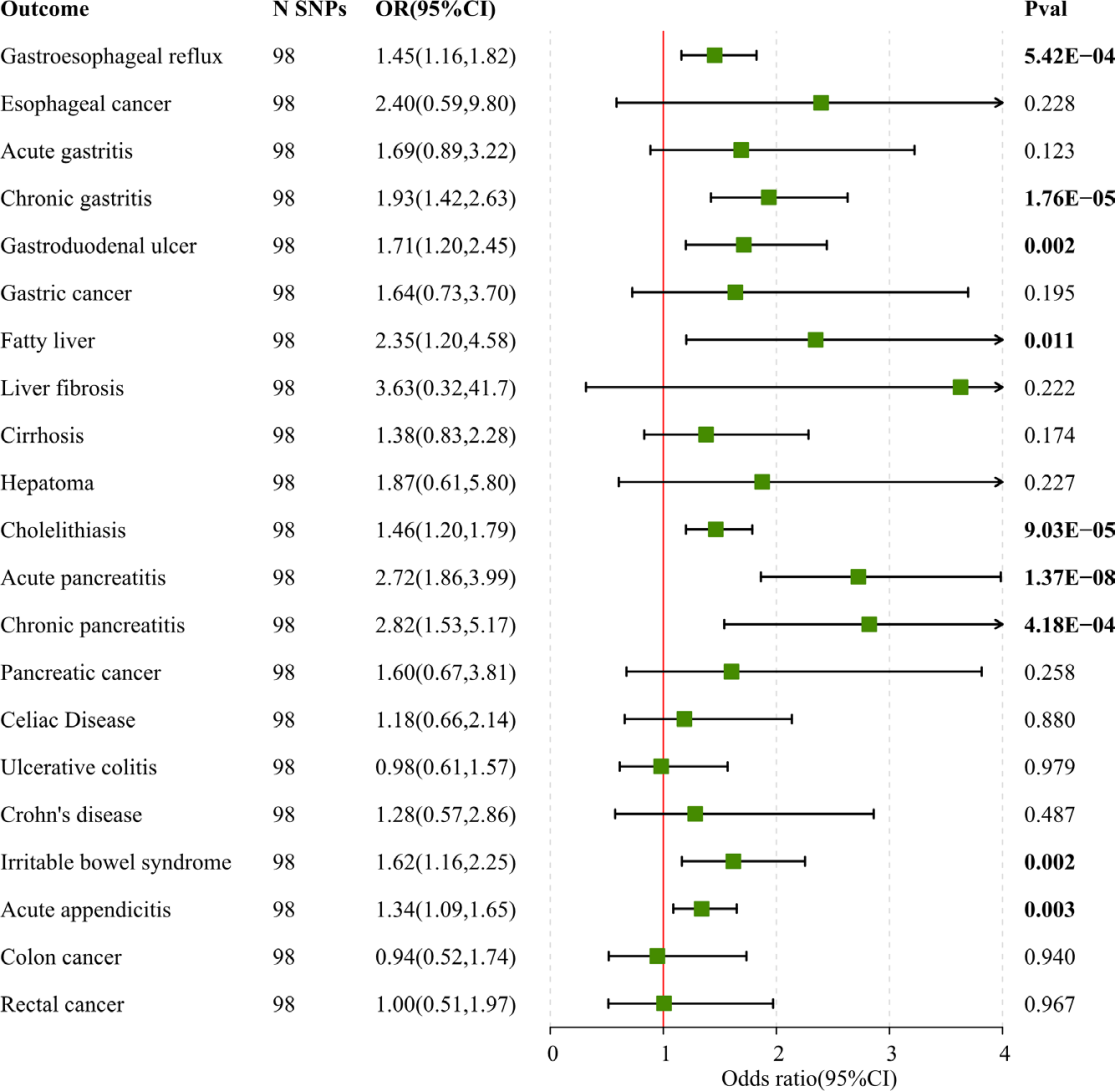
Genetic susceptibility to TV viewing time and association with 21 gastrointestinal diseases.

**Figure 2.**
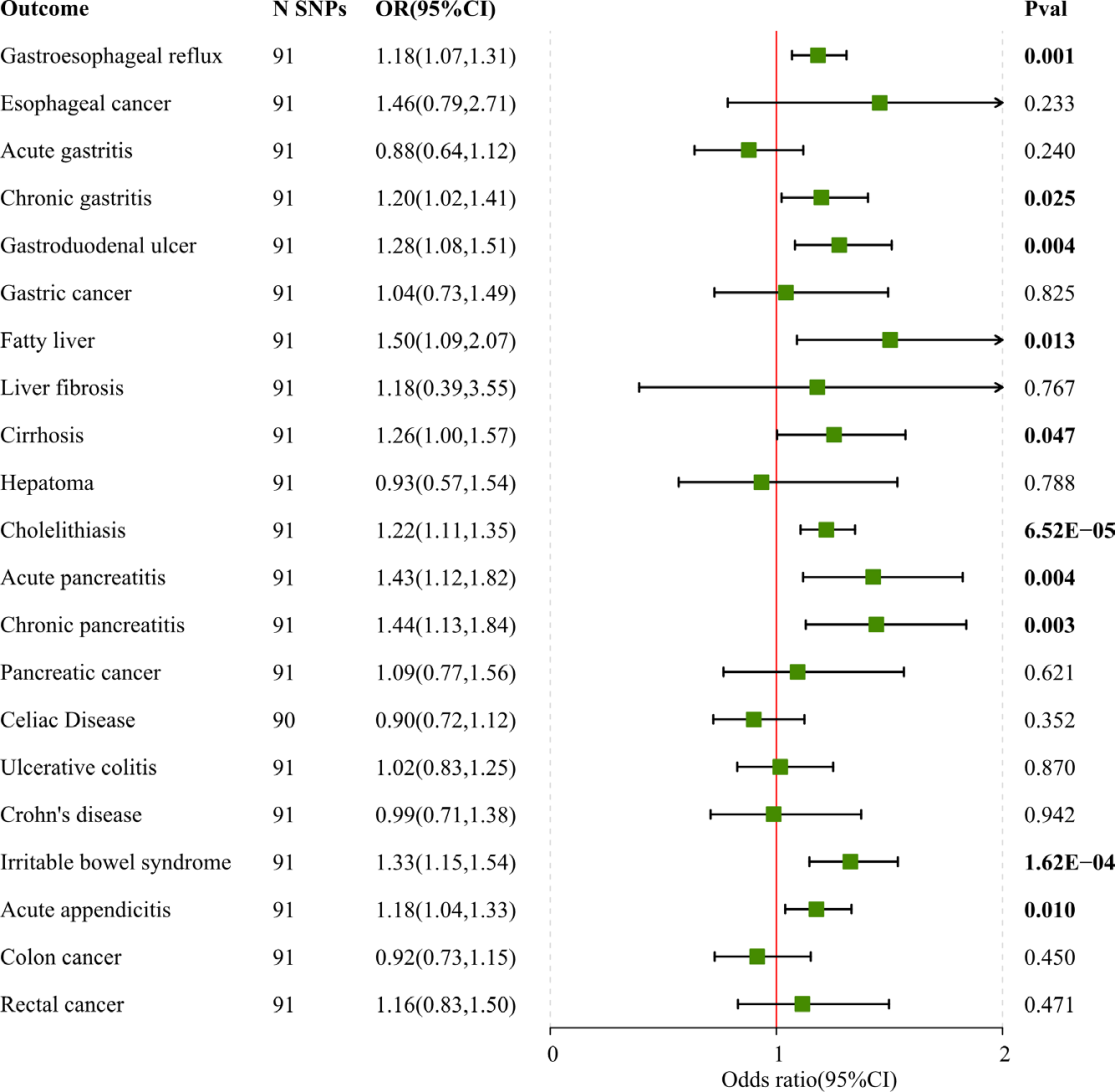
Genetic susceptibility to leisure screen time and association with 21 gastrointestinal diseases in the Validation analysis.

When conducting a univariate MR study investigating the relationship between television viewing time and gastrointestinal diseases, we observed a significant correlation between television viewing time and 9 distinct gastrointestinal diseases after applying FDR correction (Fig. 1). Specifically, genetically predicted TV watching time was associated with gastroesophageal reflux disease(GERD) (OR = 1.454; 95% CI: 1.160–1.824; *P* = 1.19E−04), chronic gastritis (OR = 1.933; 95% CI: 1.421–2.630; *P* = 2.73E−05), gastroduodenal ulcer (OR = 1.712; 95% CI: 1.199–2.446; *P* = .003), fatty liver (OR = 2.347; 95% CI: 1.203–4.579; *P* = .012), cholelithiasis (OR = 1.464; 95% CI: 1.200–1.787; *P* = 1.77E−04), acute pancreatitis (OR = 2.725; 95% CI: 1.863–3.985; *P* = 2.35E−07), chronic pancreatitis (OR = 2.821; 95% CI: 1.539–5.169; *P* = 7.90E−04), irritable bowel syndrome (IBS) (OR = 1.619; 95% CI: 1.164–2.253; *P* = .004), and acute appendicitis (OR = 1.339; 95% CI: 1.339–1.649; *P* = .005) with significant positive associations (Table S6, Supplemental Digital Content, http://links.lww.com/MD/N612). The replication analysis of LST also demonstrated consistent results with our initial discovery (Fig. 2, Table S7, Supplemental Digital Content, http://links.lww.com/MD/N612). The findings of the MR study between computer use time and gastrointestinal disorders showed evidence of a negative association between genetically predicted computer play time and GERD (OR = 0.646; 95% CI: 0.503–0.831; *P* = 6.453E−04), IBS (OR = 0.606; 95% CI: 0.408–0.902; *P* = .013) (Table S8, Supplemental Digital Content, http://links.lww.com/MD/N612). What is more, the results of our MR analysis indicated that genetically predicted driving times were not linked to gastrointestinal disorders (Table S9, Supplemental Digital Content, http://links.lww.com/MD/N612). The complementary methods MR-Egger method and Weighted median in this univariate analysis also sustain the concept of the IVW method.

## 8. Sensitivity analysis

Heterogeneity and pleiotropy in the MR analyses were assessed using Cochran’s Q test and the MR-Egger (Mendelian randomization based on Egger Regression) intercept test. In the univariate analysis, we observed slight to moderate heterogeneity and horizontal pleiotropy. In the univariate analysis, we observed mild to moderate heterogeneity and identified horizontal pleiotropy in the associations between television viewing and duodenal ulcer (*P* = .025), computer use and acute appendicitis (*P* = .020), as well as driving and IBS (*P* = .023). However, even when the MR analysis was performed again after the potential outliers were removed by MR-PRESSO, the conclusions remained unchanged (Tables S10–S12, Supplemental Digital Content, http://links.lww.com/MD/N612).

## 9. Mediation MR analysis

We analyzed genetically predicted independent influences on television watching time after adjustment for BMI, daily smoking, and diabetes to probe the pathological mechanisms between television watching and gastrointestinal disorders. The MR analysis results indicated a significant correlation between the predicted TV watching time based on Gene prediction and an increased risk of BMI, smoking, and diabetes (Table S13, Supplemental Digital Content, http://links.lww.com/MD/N612). Table 2 shows the results of multivariate MR analysis and the proportion of mediating factors. In our multivariate analysis, we observed that a substantial proportion (30.51%) of the relationship between TV watching time and gallstone was mediated by BMI. Additionally, the combined effects of BMI and 3 other mediators accounted for a considerable proportion (41.75%) of this relationship (Table 2, Table S14, Supplemental Digital Content, http://links.lww.com/MD/N612). Our discovery of genetically predicted time spent watching TV after adjustment for BMI was associated with fatty liver (OR = 1.294; 95% CI: 0.474–3.538; *P* = .616) was no longer relevant. The replication analysis unequivocally corroborates the identical conclusion (Table S15, Supplemental Digital Content, http://links.lww.com/MD/N612). In addition to this, when watching TV time was causally affiliated with 9 gastrointestinal disorders in univariate MR analysis, genetically predicted TV time was still relevant to these 9 gastrointestinal disorders, whether corrected for BMI, daily smoking, or diabetes.

**Table 2:** The association and intermediary proportion of Gene prediction TV watching time adjusted by mediators with 9 gastrointestinal diseases.

Outcome	Unadjusted	Adjust BMI		Adjust cigarettes per day		Adjust type 2 diabetes		Adjust BMI, cigarettes per day, Type 2 diabetes	
	*P*	*P*	Mediation effect (%)	*P*	Mediation effect (%)	*P*	Mediation effect (%)	*P*	Mediation effect (%)
Gastroesophageal reflux	1.19E−03	.010	7.16%	.031	10.77%	.001	11.59%	.045	19.35%
Chronic gastritis	2.73E−05	.123	0.97%	.001	4.83%	.015	10.67%	.563	15.97%
Gastroduodenal ulcer	.003	.026	2.47%	.005	10.61%	.019	12.09%	.825	27.56%
Nonalcoholic fatty liver disease	.012	.616	22.08%	.006	−7.55%	.030	21.24%	.413	32.91%
Cholelithiasis	1.77E−04	.268	30.51%	.001	13.75%	.058	8.05%	.772	41.75%
Acute pancreatitis	2.35E−07	.002	1.54%	1.11E−04	2.14%	.009	5.11%	.278	9.30%
Chronic pancreatitis	7.90E−04	.046	−5.46%	.009	5.55%	.027	11.27%	.079	16.95%
Irritable bowel syndrome	.004	.032	6.15%	.045	8.06%	.026	3.65%	.046	12.41%
Acute appendicitis	.005	9.95E−04	−2.17%	.003	7.36%	.008	3.45%	.023	10.75%

BMI = body mass index.

## 10. Discussion

In this work, we investigated the potential causal association between SB and 21 different gastrointestinal disorders using open-source GWAS databases. Our findings revealed that 9 of the gastrointestinal conditions we looked at were at risk due to watching television, a type of SB, the replication analysis also validated this finding. Both univariate and multivariate MR studies that corrected for potentially confounding variables such as BMI, smoking, and diabetes came to the same conclusions. These findings may offer important new understandings of the genetic pathways underpinning the association between gastrointestinal disorders and SB.

SB may be a substantial risk factor for more than a dozen malignancies, including esophageal, oropharyngeal, lung, endometrial, gastric, and colon cancers, according to a recent large prospective cohort study.^[35,36]^ However, no such potential association was found in the MR analysis conducted by Zhang et al and the current study.^[37]^ The disparities in the results are probably caused by the influence of prior observational research with confounding variables and unavoidable reverse causal relationships. Another reasonable explanation is that the limited sample size used in this study made it impossible to identify an association and necessitated additional research to determine the causal link between SB and cancer.

That SB promotes bad eating patterns and lifestyle choices that can eventually result in obesity is 1 explanation for the molecular linkages linking SB and cancer. As a risk factor for the development of cancer, obesity can encourage carcinogenesis and progression through a variety of pathways: altering signaling pathways and the insulin-like growth factor system, which are separate risk factors for the growth of tumor cells; ^[38]^ obese people produce more estrogen, which is linked to a higher risk of breast cancer in women after menopause;^[39]^ functioning as a persistent inflammatory biomarker that increases leptin and lipocalin release from adipose tissue, which promotes the growth of cancer by causing DNA and mitochondrial damage^[40,41]^; and dramatically raising the incidence of cancer through several routes, including adipocytokines, changed gut flora, and increased ectopic fat deposition.^[42,43]^

According to the findings of a prior prospective study looking at the relationship between physical activity and Barrett’s esophagus, being frequently sedentary is probably a risk factor for gastroesophageal reflux disease(GERD) conditions.^[44]^ Similarly to this, an MR analysis of 29,975 GERD cases showed a connection between TV viewing and computer use.^[45]^ Using MR techniques from several GWAS databases, both univariate and multivariate, the results of the current study were verified. A significant population-based cohort study further supported the link between a sedentary lifestyle and an increased risk of IBS.^[46]^ Additionally, a cross-sectional study of medical students in Peru indicated that stress and a sedentary lifestyle were independent risk factors for IBS.^[47]^ We confirmed the importance of SB as an independent factor in IBS using MR techniques. A sedentary lifestyle decreases the peristaltic action of the intestine, which can lead to decreased gravitational force for descending colon dynamics and emptying as well as chronic intestinal obstruction made worse by protracted colon distension. This is the most likely pathogenic reason for this.^[47,48]^ More studies are required to substantiate the claim that physical activity and exercise can help prevent IBS by increasing intestinal motility and absorptive capacity. Notably, this study showed that SB, such as using a computer, maybe a protective factor against IBS, but its statistical significance was not significant, and there aren’t any controlled studies that examine the relationship between the 2. So, it is advised to proceed with caution when interpreting these data.

Studies on the connection between SB and digestive disorders are scarce. The most likely explanation at this time is that SB can cause gastrointestinal issues due to a variety of reasons, including obesity, poor lifestyles, and ability intake. Adult TV consumption has been linked in numerous studies to obesity, and cutting back on TV time can result in lower calorie intake and healthier lifestyle choices.^[49,50]^ This MR study reinforces these findings.

By accounting for common risk factors for gastrointestinal disorders, this study sought to analyze the independent impact of sedentary activity while examining the relationship between SB and various gastrointestinal diseases. Gastrointestinal diseases cause significant economic losses to individuals and the nation as a whole, making it critical to raise public awareness of the harmful effects of SB,^[51]^ as demonstrated in this study. Our findings can help improve clinicians’ prevention strategies for gastrointestinal disorders and provide further guidance for patients at high risk.

The main advantage of this study is the use of MR analysis methods and the correction of BMI, smoking, and diabetes risk factors, which greatly reduces confounding factors and bias in reverse causal associations in observational studies, thereby providing accurate causal inference between SB and gastrointestinal diseases. Additionally, by restricting the GWAS population to those with European ancestry, we avoided population stratification bias. This study does, however, have certain drawbacks. First, the potential for multiplicity, which cannot be completely excluded, is the main drawback of MR analysis. To reduce the bias caused by horizontal multiplicity, we used 1-column approaches, multivariate methods, and sensitivity analysis. Second, due to the unavailability of instrumental variables estimation for gastrointestinal diseases, we were unable to investigate the 2-way association between SB and gastrointestinal diseases in the Gene prediction analysis. Third, because we used a linear approach, it is possible that we won’t be able to determine the causal link when SB and disease have a nonlinear association.

## 11. Conclusion

In summary, this MR study offers compelling evidence that limiting SB, particularly television viewing time, may be able to halt the onset and progression of some gastrointestinal disorders. Our analysis validates the hypothesis that sedentary activity has an independent impact on gastrointestinal health by controlling for common risk factors and confounding variables. Our findings imply that encouraging physical activity and minimizing SB might be a useful strategy for avoiding and managing gastrointestinal disorders, while further study is required to fully understand the underlying mechanisms.

## Acknowledgments

Thanks to the researchers who have contributed to Genome wide association study (GWAS) data.

## Author contributions

**Conceptualization:** Zhen Ding.

**Formal analysis:** Zhen Ding.

**Investigation:** Yunzhi Lin.

**Software:** Yunzhi Lin.

**Validation:** Jun He.

**Visualization:** Yunzhi Lin.

**Writing – original draft:** Yunzhi Lin.

**Writing – review & editing:** Yunzhi Lin.

## Supplementary Material


